# Hibernation of the Conduction System and Atrioventricular Block Reversibility Following Revascularization in Patients without Acute Coronary Syndrome

**DOI:** 10.19102/icrm.2023.14125

**Published:** 2023-12-15

**Authors:** Alireza Malekrah, Alireza Fattahian, Iman Majidifard, Nader Asgary, Ali Kazemisaeed, Mohamad Taqi Hedayati Goudarzi, Babak Bagheri, Aliasghar Nadi

**Affiliations:** 1 Department of Cardiology, Cardiovascular Research Center of Mazandaran University of Medical Science, Sari, Mazandaran, Iran; 2 Cardiovascular Research Center of Mazandaran University of Medical Science, Sari, Mazandaran, Iran; 3 Department of Cardiology, Kermanshah University of Medical Science, Kermanshah, Iran; 4 Department of Cardiology, Tehran University of Medical Science, Tehran, Iran; 5 Department of Cardiology, Babol University of Medical Sciences, Babol, Iran; 6 The Cardiovascular Research Center of Mazandaran University of Medical Science, Sari, Mazandaran, Iran

**Keywords:** Atrioventricular block, coronary artery disease, hibernation, pacemaker, revascularization

## Abstract

Although myocardial infarction (MI) is a reversible cause of atrioventricular (AV) block, the association of ischemia other than MI with AV block is unclear. The purpose of this study is to investigate this relationship. Among patients nominated for pacemaker implantation due to AV block in two centers from 2017–2020, 120 patients with significant coronary artery disease (CAD) in angiography were included in the study. Patients were divided into two equal groups based on their CAD treatment approach: drug therapy and revascularization. Coronary lesions were divided into three types based on location: left anterior descending artery (type 1), dominant coronary with AV node branch (type 2), and a combination of both (type 3). After coronary disease treatment, all patients were followed up with for 14 months, and AV block reversibility was assessed. There were 7 cases of block reversibility in the revascularization group (11.7%) and 1 case in the medical group (1.7%), which differed significantly (*P* = .02). A history of acute coronary syndrome, smoking, opium use, chronic kidney disease, hypertension, age, sex, and chronic obstructive pulmonary disease were not significantly associated with reversible block. Also, the type of coronary obstruction had no significant relationship with block reversibility (*P* = .3, .5, and .8 for type 1, type 2, and type 3, respectively). Hibernation due to ischemia can be a reversible cause of an AV blockage. Therefore, it is recommended that significant coronary artery lesions be revascularized before pacemaker implantation.

## Introduction

Pacemaker implantation is the only known treatment for symptomatic atrioventricular (AV) node conduction disorders. One of the most important factors in deciding to implant a permanent pacemaker is the irreversibility of conduction impairment and the presence of a persistent AV block.^1^ The known causes of AV block include abnormal blood flow supplying the conduction system and ischemia in related tissues. It is hypothesized that, if impaired coronary flow and ischemia do not lead to necrosis and fibrosis of the conduction system, AV node function may improve after revascularization. This reversibility is a well-known phenomenon in cases of acute coronary syndrome (ACS). Therefore, according to the available guidelines, after a waiting period, it can be decided whether or not to implant a pacemaker. On the contrary, the possibility of reversibility of AV block in patients with coronary artery disease (CAD) in cases other than ACS is an unknown issue for which existing clinical guidelines have not provided any recommendations.

The aim of this study is to find the answer to the question of how the conduction system behaves in cases of stable ischemia. Can this specialized system restore its function following the restoration of coronary supply and the improvement of ischemia? Moreover, in AV block (Mobitz I, II, or complete heart block) in an ischemic setting other than ACS, does coronary revascularization have practical value for block reversibility?

## Methods

Patient information in two centers from 2017 to the second half of 2020 was collected and analyzed in a retrospective and prospective combination. These patients with symptomatic AV block (symptoms ranging from light-headedness to syncope) underwent permanent pacemaker implantation after reversible causes were ruled out.

**Figure 1:**
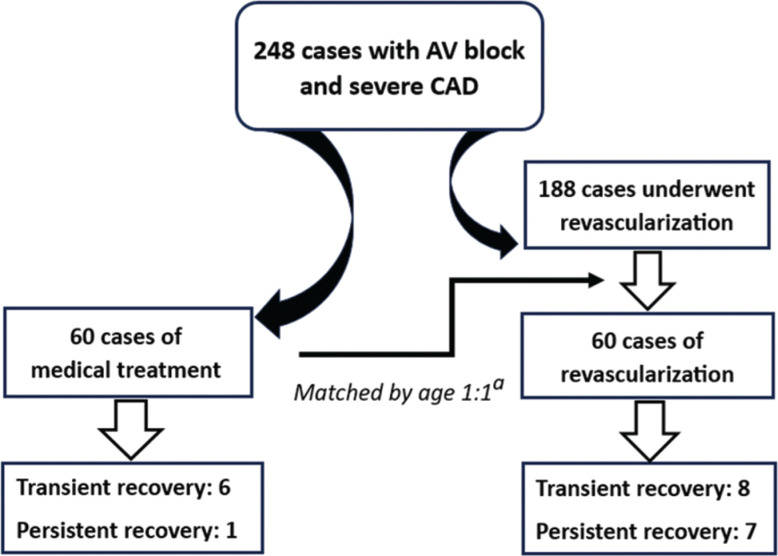
Study population. *Abbreviations:* AV, atrioventricular; CAD, coronary artery disease. ^a^Matched by age with an interval of ±5 years.

CAD in these patients was evaluated by computed tomography angiography, myocardial single-photon emission computerized tomography, or coronary angiography, and those who showed significant coronary obstruction with the first two methods underwent coronary angiography for a definitive diagnosis. Of these, 248 cases had severe CAD, including 60 who received medical treatment, while the rest underwent revascularization with percutaneous coronary intervention (PCI) or coronary artery bypass grafting (CABG). Each patient in the medical group was matched with a patient in the revascularization group in terms of age with an interval of ±5 years **(Figure 1 and Table 1)**.

Reasons for depriving the medical group of revascularization included patient preference, unsuitable coronary artery anatomy, or the physician’s decision to prioritize medical treatment. Patients who did not undergo coronary evaluation, who had insignificant CAD or significant coronary disease unrelated to the conduction system, or in whom AV block occurred in the ACS setting were excluded from the study. Also, patients implanted with an implantable cardioverter-defibrillator due to an ejection fraction of <35% were not included in the study.

**Figure 2:**
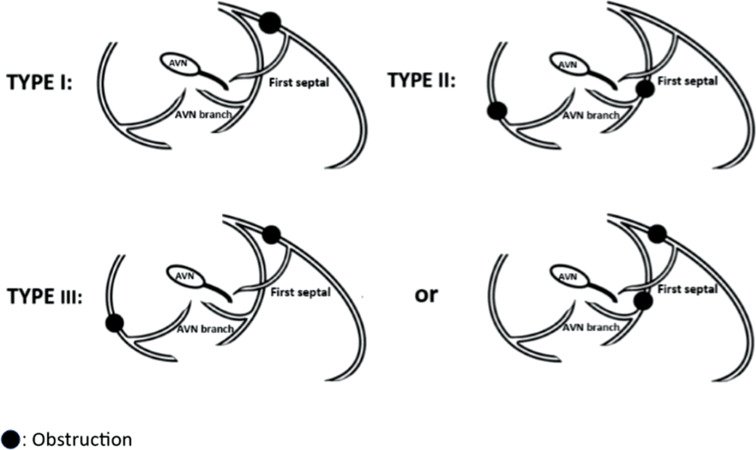
Types of coronary artery obstruction. *Abbreviation:* AVN, atrioventricular node.

Patients with CAD, according to the location of the lesion, compromising blood supply to the AV node and bundle of His were assigned to three groups, as follows **(Figure 2)**:

- Type I: Significant obstruction in the left anterior descending artery (LAD), proximal to the first septal branch
- Type II: Significant obstruction of the AV node branch or proximal to it (of the dominant right coronary artery [RCA] or left circumflex artery [LCX])
- Type III: Significant obstruction in both the LAD, proximal to the first septal branch, and the AV node branch or proximal to it (of the dominant RCA or LCX)

A visually estimated diameter stenosis severity of ≥70% was defined as significant stenosis.^2^

Although it is theoretically possible that the AV nodal branch originates from the hyper-dominant LAD and that AV block occurs due to LAD occlusion other than the proximal part, this particular anatomy was not present in our study and therefore was not included in this classification.

After coronary evaluation and initiation of medical treatment or revascularization by PCI, without a waiting period (according to available guidelines), on average 2 days later, a single- or dual-chamber pacemaker, depending on the patient’s condition, was implanted. Patients treated with CABG were either implanted with an epicardial pacemaker during surgery or had an endocardial pacemaker implanted during hospitalization. The mean age of the patients at pacemaker implantation was 68.7 years, and the age range was 47–79 years. A pacemaker analysis was performed at the time of discharge from the hospital, 1 week and 1 month after discharge and then every 5 months thereafter. If AV conduction reversibility was observed, a re-analysis was performed 1 week later. Cases with normal and stable AV node function in at least two separate analyses without any recurrence until the end of the study were considered cases of reversible block.

Based on the assumption that restoration of hibernation tissue function may occur up to 14 months after surgery,^3^ patients were followed up with for at least for the same period, and reversibility cases were recorded as the improving effect of revascularization on the AV block.

This study was conducted at Mazandaran University of Medical Sciences in Sari, Iran. The study was approved by the ethics committee of Mazandaran University of Medical Science, and written consent was obtained from the enrolled patients.

### Statistical analysis

Categorical variables are expressed as frequencies and percentages. Continuous variables with normal distribution are shown as mean ± standard deviation values. Student’s *t* test was used for the comparison of normally distributed continuous variables, and the chi-squared test was used for categorical variables. *P* < .05 was considered statistically significant for all tests. Data analyses were performed using SPSS V.24 software (IBM Corporation, Armonk, NY, USA).

## Results

Angiographic findings showed that type II lesions were the most common lesion type among the medical and revascularization groups. In the revascularization group, 8 patients underwent CABG, and the remaining 52 underwent coronary angioplasty. Three of the CABG patients underwent epicardial pacemaker implantation during surgery, and an endocardial pacemaker was implanted in the other 5 cases after surgery. The angiographic findings of the patients are summarized in **Table 2**.

According to the schedule, patients were followed up with for at least 14 months after implantation of the pacemaker. During the follow-up period, 8 patients in the vascularization group and 6 patients in the medical treatment group showed transient block reversibility. These were cases of unstable recovery in which block recurrence was recorded again during the follow-up period.

**Figure 3:**
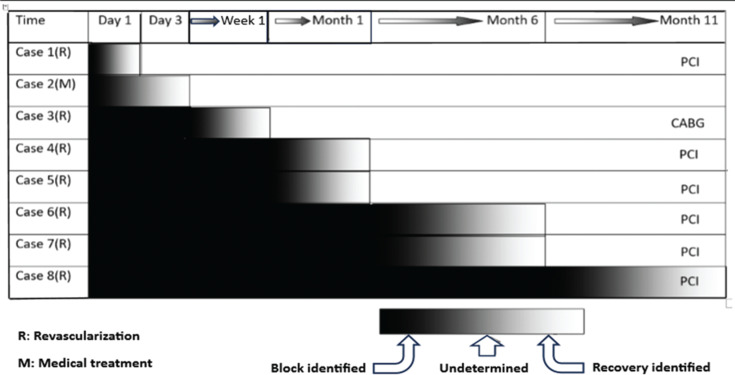
Timetable of recovery. *Abbreviations:* CABG, coronary artery bypass grafting; PCI, percutaneous coronary intervention.

After exclusion of transient reversibility, 8 patients regained their AV node function persistently **(Table 3)**, including 1 patient belonging to the medical group and 7 belonging to the revascularization group, which showed a significant difference between the two groups (*P* = .02). Two cases of reversibility occurred during hospitalization and before the implantation of a pacemaker, one of which belonged to the medical group. A 14-month follow-up analysis of both patients showed normal and stable functioning of the AV node. Block reversibility was detected in another 6 cases after pacemaker implantation and in analysis sessions. The shortest reversibility time of AV function after revascularization was 1 day and the longest was 10 months, respectively **(Figure 3)**.

The results show no significant relationship between the reversibility of AV node function and variables of age, sex, history of ACS, opium or opioid use, smoking, hypertension, chronic kidney disease, and chronic obstructive pulmonary disease **(Table 4)**. Also, AV block reversibility had no significant relationship with the location of coronary obstruction and the type I, II, and III subgroups.

## Discussion

The study’s main finding was that, in patients with AV block and non-ACS conditions, revascularization of the coronary arteries supplying the AV node and His could significantly cause AV block reversibility.

Historically, the relationship between AV node blood flow disturbance and the occurrence of AV block has been known. For the first time, Bassan et al. showed that, even in patients with inferior wall infarction, the presence of an obstruction in the LAD increases the risk of AV block by six times compared to that in cases where there is only an obstruction in the RCA.^4^

The “Bassan” hypothesis was based on the findings by Kennel and Titus. Their anatomical studies had shown that, in the hearts of 80% of humans, there is an anastomotic connection between the AV node artery and the first septal branch of the LAD.^5^ Accordingly, in this study, all coronary disorders that could lead to conduction disturbances were included in the study by dividing coronary disorders into three types.

Reversibility of AV block after revascularization in patients with obstructive CAD is a topic that is less discussed in existing articles. One of the most important studies by Hwang et al. examined 188 patients with AV block, including 58 with severe CAD. In this study, only 2.3% of patients with stable angina recovered from AV block. Their study showed that AV block is irreversible in cases other than those with myocardial infarction (MI).^6^ The results of their study are not in line with those of our study; the main reason is that Huang et al. only compared two groups of acute MI and non-acute MI and did not study the effects of revascularization as a variable.

In another important study, Yesil et al. specifically studied the effect of revascularization on block reversibility. They assigned 16 patients to the medical group and 37 patients to the revascularization group and followed them for 36 ± 6 months. AV block remained persistent in 13 patients in the medical group and 27 in the revascularization group, which showed no significant difference.^7^ The results of this study are also different from ours. They may be different due to the small number of samples and exclusion criteria. On the contrary, the number of reversibility cases in their study was 12, which is more than that in our study. This may be due to the inclusion of transient reversibility cases or a longer follow-up period. Our study recorded 8 cases of transient reversibility in the revascularization group and another 6 cases in the opponent group. This transient reversibility cannot be attributed to transient ischemia because it occurred more often in the first group, where the ischemia had been resolved. The most likely explanation for this phenomenon may be the effect of autonomic fluctuations on the diseased AV node, as we had excluded other potential causes.

The rest of the studies in this area have been case studies that cannot be cited for conclusions. Omeroglu et al. studied 8 patients with AV block and severe CAD. All of these patients underwent CABG, and no cases of AV block reversibility were recorded.^8^

Narin et al. also reported 2 cases of patients with AV block who underwent CABG. One patient had a persistent block and was implanted with a pacemaker, but the other patient recovered immediately after revascularization.^9^

## Possible causes of atrioventricular block reversibility

### Non-ischemic causes of reversibility

The most important non-ischemic causes of reversibility are β-blockers, calcium channel blockers, and anti-arrhythmic drugs, as well as causes such as myocarditis or hypothyroidism. Because all of these causes were ruled out before pacemaker implantation and drugs were discontinued for at least five half-lives, reversibility could not be attributed to these causes.

### Ischemic causes of atrioventricular block reversibility

#### Inflammatory mediators and autonomic tone alteration

Increased vagal tone is a known cause of transient AV blocks in acute MI (AMI), especially in inferior MI. The widespread distribution of cardiac receptors along the vagal afferent pathways in the inferior wall and their stimulation by ischemia cause Bezold–Jarisch phenomenon, which presents with bradycardia and AV block. Although Bezold–Jarisch phenomenon has been reported in cases of ischemia without MI, its transient nature may not justify a persistent block in the patients in our study.

Another possible mechanism apart from the vagal tone is the release of inflammatory mediators, including potassium and adenosine, which are released as a result of myocardial ischemia.^10^ AV block is a well-known phenomenon as a result of hyperkalemia, and myocardial ischemia can increase potassium levels within a few seconds by releasing from myocytes and disrupting extracellular washout.^11^ As acute ischemic hyperkalemia, which leads to transient AV block, has been only reported in AMI, it cannot be a justification for such an AV block that occurs without AMI and persists for several months even after revascularization. By the same explanation, the effect of adenosine—which, like potassium, is released from myocytes in acute ischemia—cannot be generalized to such a block.

Although the chronic increase in extracellular adenosine unrelated to ischemia has a known effect in some diseases, such as skin diseases, liver disorders, and chronic kidney and lung diseases, these disorders are mainly associated with the stimulation of receptors A (2A) and A (2B),^12^ and there are no reports of AV block due to chronic stimulation of receptor A (1), which is the dominant receptor in the node. Even if we propose it as a hypothesis that chronic adenosine signaling stimulates receptor A (1) and causes AV block, no justification can be given for improving the block after revascularization.

#### Hibernation

Myocardial hibernation is a well-known phenomenon with two main characteristics. The first is myocardial contractile dysfunction resulting from resting ischemia, and the second is dysfunction’s reversibility after establishing the coronary flow. Although much is known about myocardial hibernation, it is not a well-known phenomenon in the conduction system. The presence of both main features of hibernation in AV reversible block (ischemia and recovery following coronary flow establishment) strongly suggests that the hibernation mechanism has a similar function in the cardiac conduction system as a surviving force. Improved AV node function over 1 day to 10 months indicates that, like the myocardium, both short-term and chronic hibernation occur in the conduction system.

Based on these findings and the behavior of the AV conduction system in response to ischemia and after coronary flow recovery, the most probable hypothesis to justify the results of this study is the occurrence of hibernation in the conduction system.

## Limitations

One of the main limitations of this study is the diagnosis of ischemia based only on anatomical and angiographic criteria. Anatomical findings cannot be a precise indicator of ischemia, and a complete study with functional tests can provide more reliable results.

Although hibernation is considered the most probable cause of block reversibility based on the results and behavior of the conduction system, additional studies at the cellular and molecular levels as well as AV node cell physiology in chronic ischemia and post-revascularization status could provide better information in this regard.

## Conclusion

According to this study, in cases of AV block associated with significant obstruction in the coronary supplying the AV node, coronary revascularization can cause AV block to recover. Therefore, it is recommended that, for patients with AV block requiring a pacemaker, a coronary evaluation be performed before pacemaker implantation. In the case of severe coronary obstruction supplying the node, revascularization should be performed. As only two cases of block reversibility occurred during hospitalization, it is not recommended to delay pacemaker implantation pending block recovery.

## Figures and Tables

**Table 1:** Patient Characteristics According to Group

Variables	Medical Group	Revascularization Group	*P* Value
Mobitz I	19	21	.7
Mobitz II	12	8	.3
CHB	29	31	.7
Male sex	33	29	.4
History of ACS	22	28	.2
Smoking	26	20	.3
Opium or opioid	6	10	.3
CKD	2	1	.6
COPD	2	5	.051
HTN	28	38	.06
Age (years)	67.6 ± 8	69.8 ± 6	.09

*Abbreviations:* ACS, acute coronary syndrome; CKD, chronic kidney disease; COPD, chronic obstructive pulmonary disease; HTN, hypertension

**Table 2:** Angiographic Characteristics of Patients

Lesion Type	Medical Treatment	Revascularization
Type I	16	10
Type II	35	40
Type III	9	10
RCA dominancy	46	51

*Abbreviation:* RCA, right coronary artery.

**Table 3:** Electrocardiogram Features in Recovered Patients (Before Recovery)

2:1 AV block with narrow QRS	Case 4
Advanced heart block with wide QRS (fixed 3:1 block)	Case 2
Advanced heart block with wide QRS (different ratio block)	Case 8
Complete heart block with junctional escape	Cases 1 and 5
Complete heart block with ventricular escape	Cases 3, 6, and 7

**Table 4:** Variables Associated with Atrioventricular Block Recovery

Variables	Reversibility	Non-reversibility	*P* Value
Revascularization/medical	7/1	53/59	.02
CABG/PCI	1/6	7/46	.9
Revascularization of type I	2	8	.3
Revascularization of type II	4	36	.5
Revascularization of type III	1	9	.8
Male sex	4	58	.9
History of ACS	3	47	.8
Smoking	4	42	.4
Opium or opioid	2	14	.3
CKD	1	2	.06
HTN	3	63	.3
COPD	1	6	.4
Age (years)	67.6 ± 5	68.75 ± 7	.7

*Abbreviations:* ACS, acute coronary syndrome; CABG, coronary artery bypass grafting; CKD, chronic kidney disease; COPD, chronic obstructive pulmonary disease; HTN, hypertension; PCI, percutaneous coronary intervention.
